# Safety Needs Mediate Stressful Events Induced Mental Disorders

**DOI:** 10.1155/2016/8058093

**Published:** 2016-09-21

**Authors:** Zheng Zheng, Simeng Gu, Yu Lei, Shanshan Lu, Wei Wang, Yang Li, Fushun Wang

**Affiliations:** ^1^School of Psychology, Nanjing University of Chinese Medicine, Nanjing 210023, China; ^2^School of Psychology, Nanjing Normal University, Nanjing 210023, China; ^3^Nanjing University of Chinese Medicine, Nanjing, China; ^4^School of Psychology, Nanjing University of Forest Police, Nanjing 210023, China

## Abstract

“Safety first,” we say these words almost every day, but we all take this for granted for what Maslow proposed in his famous theory of* Hierarchy of Needs*: safety needs come second to physiological needs. Here we propose that safety needs come before physiological needs. Safety needs are personal security, financial security, and health and well-being, which are more fundamental than physiological needs. Safety worrying is the major reason for mental disorders, such as anxiety, phobia, depression, and PTSD. The neural basis for safety is amygdala, LC/NE system, and corticotrophin-releasing hormone system, which can be regarded as a “safety circuitry,” whose major behavior function is “fight or flight” and “fear and anger” emotions. This is similar to the Appraisal theory for emotions: fear is due to the primary appraisal, which is related to safety of individual, while anger is due to secondary appraisal, which is related to coping with the unsafe situations. If coping is good, the individual will be happy; if coping failed, the individual will be sad or depressed.

## 1. Maslow's Hierarchy of Needs Revisit

**Figure 1:**
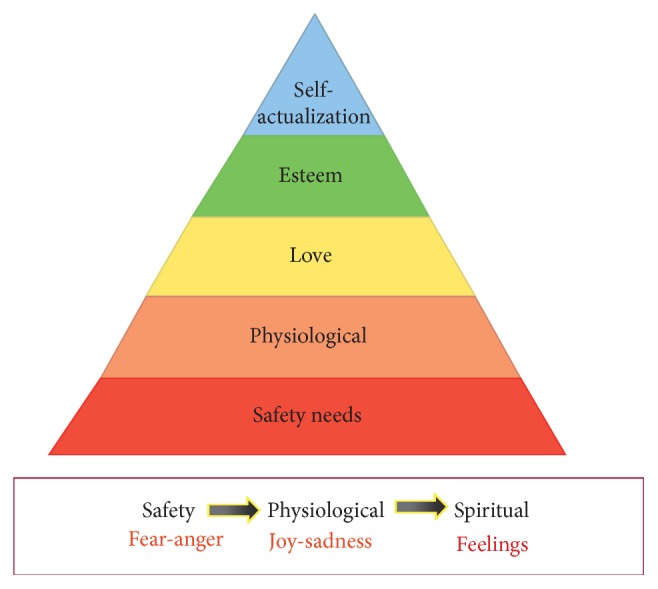
New version for* Hierarchy of Needs*: “*safety-hedonic-esteem-love-self-actualization*”. All emotions and feelings depend on whether the stimulus can satisfy the needs of individuals, and the satisfaction of different needs can induce different emotions: safety can induce fear and anger, hedonic needs can induce joy and sadness, and spiritual needs can induce feelings such as love.

Maslow's Hierarchy of Needs has been a well-known theory since Abraham Maslow proposed it in 1943 [1] and in his book* Motivation and Personality* in 1954, in which he proposed that human needs can be portrayed in the shape of a pyramid, with the most fundamental levels of needs at the bottom. From bottom to the top are the needs:* physiological needs, safety, belongingness and love, esteem, and self-actualization*. Physiological needs are the physical requirements for the survival of individual and the animal kind, such as food and sex, and safety needs are personal security, financial security, and health and well-being. Maslow proposed that if the physiological needs are relatively satisfied, there then emerges a new set of needs, which may be categorized roughly as the safety needs. The organism may be wholly dominated by them, which may serve as the almost exclusive organizers of behavior, recruiting all the capacities of the organism in their service, and we may then fairly describe the whole organism as a safety-seeking mechanism [1]. But Maslow also found that “practically everything looks less important than safety, even sometimes the physiological needs which being satisfied, are now underestimated. A man may be characterized as living almost for safety alone.” [1]. Actually safety needs are more fundamental than physiological needs. Safety needs are personal security, financial security, and health and well-being, which are more fundamental than physiological needs (Figure 1). For example, the deer cannot eat (physiological needs) on the wild prairie when the wolves chase them (safety). Let me take one example from Cosmides and Tooby (2000), who propose that the emotions serve to regulate behavior. He wrote about fear like this: “Imagine walking alone at night and hearing some rustling in the brush. Your energies are aroused to be ready for action, you become acutely aware of sounds that could indicate that you are being stalked, the threshold for detecting movements is lowered, you no longer feel pangs of hunger, attracting a mate is the farthest thing from your mind.” [2]. So it is clear that safety needs are more fundamental than physiological needs. Whether physiological needs or safety needs are more important will affect our opinions about mental disorders, for example, Freud proposed libido (physiological needs) more important.

**Figure 2:**
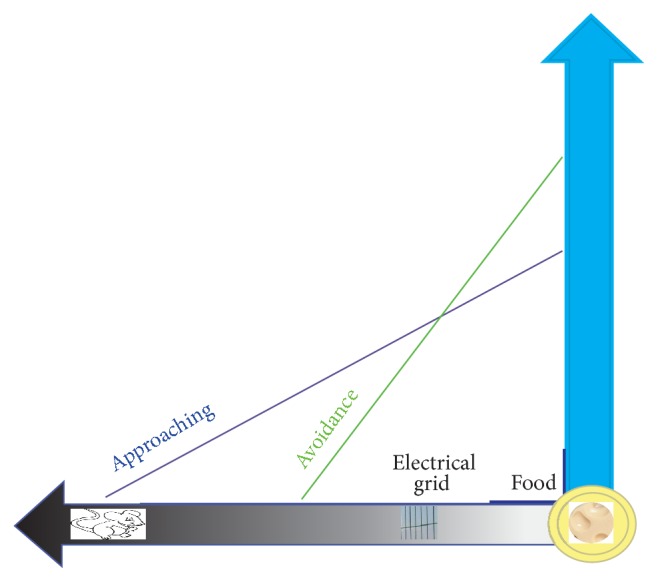
Safety needs can block physiological needs. Approach and avoidance gradients depend on the distance from the goal. Goal includes the food (physiological needs), while the punishments include the foot shock (safety needs). The avoidance slope is steeper and predominates proximally to the goal, whereas the approach gradients are higher at the remote location.

*Safety Needs Can Block Physiological Needs.* Safety needs can block physiological needs; the classical example is Miller's Avoidance and Approach experiments (Figure 2) [3]. In 1961, Neal Miller used behavioral measures to assess motivational disposition in rodents (Figure 2). As the animal moves closer to the potential reward (e.g., food), the force exerted to obtain the reward increases. Similarly illustrated is the avoidance: the force the animal exerted to avoid the aversive stimulus (a shock) also increases as the animal comes closer, and furthermore, the slope of the avoidance gradient tended to be steeper than that of approach [3]. Later experiments by Ito found that the organisms tend to be more sensitive to the threatening information and generally process such information faster than the rewarding information [4]. He called this phenomenon negativity bias and attributed it as a protective strategy through evolution, since even a single failure to respond adaptively to a survival threat may preclude passing on genetic information. He found that “as a potential treat looms, the adaptive response of the brain is to amplify these threats and initiate appropriate behavioral responses, such as fleeing, freezing, or attacking.” And he found that the negativity bias can be seen across all levels of the neuraxial organization [3]. These data support the notion that safety needs are faster and more fundamental than hedonic needs.

## 2. Safety Needs—Unexpectancy

Maslow thought adults usually inhibit the reaction for safety needs, so he used infants as an example and found that the child's need for safety is his preference for some kind of undisrupted routine or rhythm. Maslow mentioned, “He seems to want a predictable, orderly world. For instance, injustice, or inconsistency in the parents seems to make a child feel anxious and unsafe. This attitude may be not so much because of the injustices per se or any particular pains involved, but rather because this treatment threatens to make the world look unreliable, or unsafe, or unpredictable.” [1]. Maslow also mentioned, “Confronting the average child with new, unfamiliar, strange, unmanageable stimuli or situations will too frequently elicit the danger or terror reactions.” Therefore the safety is related to unexpectancy.

This is consistent with the behaviorists, who propose that behavior is a process-form “Stimulus-Reaction”, while cognition scientists extend it to “Stimulus-Opinion-Reaction.” However, they all depend on a stimulus. Everything around us is stimulus that has the* hedonic value*, which fits into our personal physiological needs. And it also has another feature: happening in an expected way, or unexpected way, which is related to threat and can be called* safety value*. We draw these two features of stressful events in two dimensions:* hedonic value and safety value* (Figure 1). The safety value has nothing to do with the hedonic value, for both the liked things and disliked things can induce unsafety. For example, we worry about losing the liked things and also worry about getting the disliked things; and we also will not be angry if we lose the good thing as expected and also get the disliked thing as expected. Even though it is something you liked, if it is unexpected, you still feel afraid and angry.* Therefore the safety is related to unexpectancy*. One feature of the safety need induced emotion is the rapid detection of potential threats and can initiate appropriate approach/avoidance behaviors.

*Prediction Error.* The most interesting and influential line of empirical and theoretical work is predication error [5]. The studies were done from electrophysiological recordings of dopamine neurons in awake, behaving monkey in Schultz's lab. The recordings showed that the firing of the dopamine cells only related to “prediction error” [6]. These phased activation does not discriminate different types of rewarding stimuli [6]. And it is quite unexpected that the reward delivery will not elicit dopamine neuron firing, once the animal has learned the stimulus and reward association [6]. Therefore, dopamine neurons are related to expectation about external stimulus rewarding, especially when it is uncertain or prediction error [6, 7].

## 3. Emotion Flow

**Figure 3:**
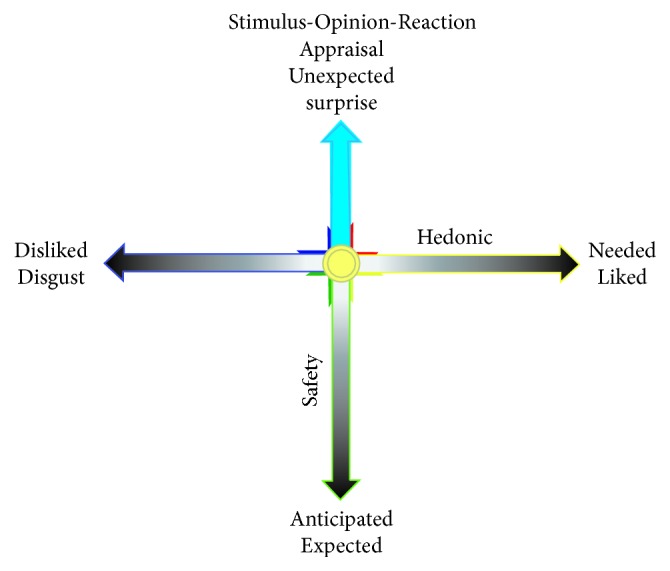
*Stressful events are something that happened unexpectedly*. Every stimulus has the* hedonic value* (horizontal dimension), which fits into our personal needs (pleasant things, needed things, or disliked things or unpleasant things). And it can happen in an expected way, or unexpected ways (vertical dimension), which are called* safety value*.

The studies of emotions have been expanded exponentially by two prominent researchers: Magda Arnold and Richard Lazarus, who proposed Appraisal theory. Appraisal theory states that emotions result from people's interpretations and explanation of their circumstances. In the structural model of Appraisal theory, Lazarus borrowed the concept of appraisal from Arnold and elaborated the concept as a key factor for emotions: emotional processes depend on the predictability of the stressful events. He distinguishes two basic forms of appraisal, primary and secondary appraisal [8], and he proposed that the primary appraisal and its induced emotions are a faster activating, automatic process, which is similar to the safety need. Indeed, Lazarus distinguishes three types of stressful events: harm, threat, and challenge, which are related to primary appraisal. The secondary appraisal is concerned with coping options, which include blame or credit, coping potential, and future expectations.* It seems that the primary appraisal is related to fear and the secondary is related to anger* (Figure 3). Lazarus mentioned that if a person appraises a situation as motivationally relevant, motivationally incongruent and also holds a person other than himself accountable, the individual would most likely experience anger in response to the situation.

Process model of the Appraisal theory is more accurate to explain the safety needs, for personal safety, which are related to the “unexpected ways of stimulus occurring.” The process model proposed two main appraisal processes: perceptual stimuli and associative processing and reasoning. Perceptual stimuli are what the individual picks up from his surroundings, such as sensation of pain or pleasure. Then, the individual performs two main appraisal processes: associative processing, which is memory based, and reasoning, which is a slower and more deliberate process that involves logical thinking about the stimulus.

All stressful events will first induce fear and anger [9]. For example, when you meet a car that quickly passes and stops before you, you will be firstly scared and then blame the car. Let us take another example from Izard's paper [10],* “when Rafe was hit from the back by a wheel chair, the first reaction of him was scared and angry, and showed angry expression and clenched fist. But after he turned back to see Rebecca, a person with hemiplegia whose wheelchair had gone out of control and cause her to crash into Rafe. Rafe's understanding changed his anger to sadness and sympathy.”* So when something unexpected occurs, you will first evaluate its threat (fear/anger) and next evaluate its hedonic value (happy/sad) (Figure 3). Similar* emotional flow* happens in our lives all the time: everything in our lives is normally calm as expected; but you will first feel scared (fear) when something unexpected occurs, and then you will blame (anger) the un-expectancy after fear is gone. And afterwards you might feel happy after successfully coping with the stressful events or feel sad if you failed to cope with them. Finally, the stressful events go away, and people calm down. This kind of emotional flow, big or small, long or short, constitutes our everyday emotions. So* fear-anger-happiness-sadness-calm* might constitute the rainbow of emotions or* emotional flow* in our everyday life.

## 4. Neural Substrate-Amygdala

The amygdala has been proved to be the neural basis for fear [11], and it is also recognized as the neural basis for stress elicited fear and anxiety [12, 13]. In addition, electrical stimulation of the amygdala promotes autonomous reactions and stress-like behavioral, whereas amygdala ablation induced a marked tameness increase, motivation loss, and fear decrease to aversive stimuli [14, 15]. Amygdala is one of the most important limbic structures that link to fear, which was first suggested by Klüver & Bucy in 1937, who demonstrated that the lesion of the medial temporal lobe resulted in a wide range of odd behaviors, such as approaching normally to fearful objects [16]. And about 20 years later, Weiskrantz (1956) found that it is the amygdala whose impairment resulted in the odd behaviors, which are called Klüver and Bucy syndrome [15]. These patients with amygdala impaired failed to learn conditioned fear responses. LeDoux puts amygdala as the emotional computer to work out the emotional significance of stimuli [17]. He demonstrated two neural pathways of sensory information from the thalamus to the cortex: (1) A slow-acting* “thalamus-to-cortex circuit,”* whose function is to analyze sensory information in detail, and (2) a fast-acting* “thalamus-amygdala circuit,”* whose major function is to analyze simple stimulus features, which bypass the cortex [18]. These two pathways possibly underlie the two evaluation systems:* the fast one for the fear/anger and the slow one for hedonic*. Other reports, such as findings from Ohman and Soares (1994) also support a fast-acting system for threat detection that involves only minimal cortical processing [19, 20]. In addition, Morris et al. (2001) also reported a patient whose primary visual cortex was impaired and therefore showed no conscious visual perception but showed significant fearful reports [20]. In all, the fast-acting thalamus-amygdala circuit is important for our ancient ancestors to rapidly recognize dangers to help with survival.

## 5. NE-Safety Neuromodulators

**Figure 4:**
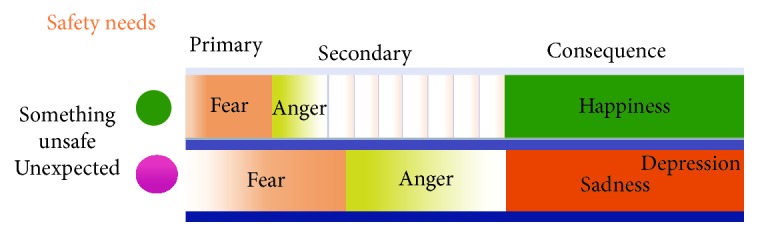
*Emotional flow*. Lazarus's primary appraisals are for threads, while secondary appraisals are for coping. Safety needs induce stressful emotions, fear and anger, and stressful behaviors, fight or flight. So fear-anger-happiness-sadness-calm constitutes the* rainbow of emotions or emotional flow* in everyday life. The figure is adopted from our previous publication [30].

It is an evolutionary adaptation for our ancestors to better cope with the unexpected environment with the fast activing thalamus-amygdala circuit. In addition to amygdala, the NE/LC system is important to direct behaviors of the animal to cope with the dangerous environment, and the well-known function of NE/LC system is to induce “fight or flight” behaviors. Take a deer in the wild as an example. When a deer meets a lion, the reaction of the deer is flight (fear), while the reaction of a lion is fight (anger). So the same neurotransmitter NE might undergo two different behaviors. So the emotion fear and anger might also be derived from the same neurotransmitter NE and the same stressful event (Figure 4). NE is released from the locus coeruleus (LC) in the brain to keep the brain alert, which has been described as increasing the “signal to noise” of the sensory inputs [21]. Many reports have implicated LC in alertness, anxiety [22–24]. And LC has been reported to be activated at stressful events: increased LC neuron firings were observed at visual threat [25]. And LC stimulation can induce fearful behaviors [26]. LC sends projections to amygdala [27], which is the most important limbic structure that links to fear [13]. Therefore, amygdala and NE/LC system might constitute the neural structure for safety needs.

**Figure 5:**
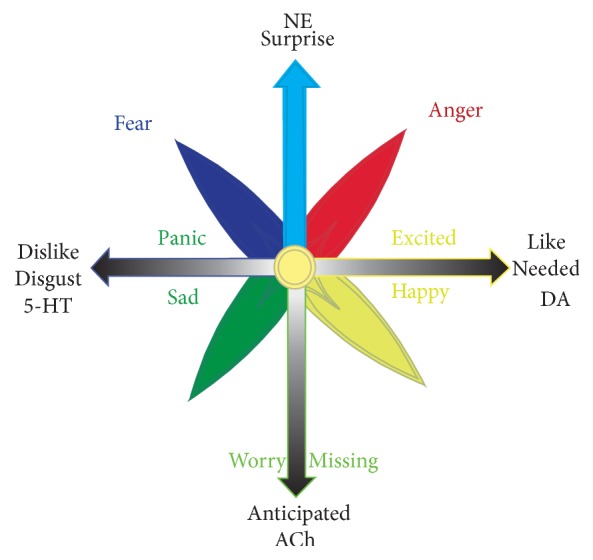
*Emotions are due to the hedonic value of the things (physiological needs) and also the way things occur (safety needs)*. The way everything occurs not only induces stressful emotions but also affects the tension of hedonic emotions. The neurotransmitters for these emotions are dopamine (a pleasant marker) and serotonin (unpleasant marker). NE is surprise marker, while Ach is a marker for anticipation. NE is well known for fight (anger) or flight (fear) behaviors.

Our ancestors navigating rich environments had to face very complicated environments, with many forms of uncertainties [28]. So they evolved an adaptive mechanism to first do a safety check for everything around them. If it would happen in an anticipated way, they will feel calm; instead if something happened surprisingly, they will be scared and angry. Therefore,* fear and anger are due to things happening in an unexpected way *(Figure 4). Of note, the unexpectancy will also increase the tension of hedonic emotions. With the same kind of hedonic stimulus, if it comes in an unexpected way, people will feel excited; instead, people will feel happy. It is the same with sadness or helpless panic (Figure 5).

## 6. Pathological Conditions

Safety needs can induce diseases, as Maslow mentioned, “One reason for the clearer appearance of the threat or danger reaction in infants, is that they do not inhibit this reaction at all, whereas adults in our society have been taught to inhibit it at all costs. Thus even when adults do feel their safety to be threatened we may not be able to see this on the surface. Infants will react in a total fashion…In infants we can also see a much more direct reaction to bodily illnesses of various kinds… for instance, vomiting, colic or other sharp pains.” [1]. Even the adults can inhibit our reactions, they still can react in some mental disorders. Maslow wrote, “Some neurotic adults in our society are, in many ways, like the unsafe child in their desire for safety, although in the former it takes on a somewhat special appearance. Their reaction is often to unknown, psychological dangers in a world that is perceived to be hostile, overwhelming and threatening.” The neurotic individual may be described in a slightly different way with some usefulness as a grown-up person who retains his childish attitude toward the world. This is to say, a neurotic adult may be said to behave as if he were actually afraid of a spanking, or of his mother's disapproval, or of being abandoned by his parents, or having his food taken away from him. It is as if his childish attitude of fear and threat reaction to a dangerous world had gone underground, and untouched by the growing up and learning processes, were now ready to be called out by any stimulus that would make a child feel endangered and threatened [1].

*Fear-Phobia.* If something happens surprisingly, people will be scared and angry; and if it would happen in an anticipated way, they will feel calm. So for the phobia patients, their problems might be that they cannot successfully accomplish the emotional flow (*fear-anger-happiness-sadness-calm*). The best way to remove fear is anger, these patients are too timid to show anger, so their emotions are checked at emotional flowing from fear to anger. Therefore, anger might be the best treatment for these patients and NE is the neural substrate for them.

*Anger-Depression.* Depression is characterized by unrelenting sadness accompanied by an inability to derive pleasure from positively hedonic situations. Therefore, depression might be related to the primary appraisal, the worrying about safety instead of physiological satisfaction. Indeed, excessive self-blame and feeling worthless are symptoms of major depression episodes across cultures [29], which is similar to Lazarus's secondary appraisal. So the depressed patients have problems with anger or with coping appraisal or their problem is due to inability to cope with the unsafe stressful situation and showed inward anger. The difference between fear and anger is the direction of the behavior: fear is to throw oneself away from the stimulus and anger is to throw the stimulus away. Depression is the inward anger. Anger is usually fight against the outside stimulus. For these patients, they do not have the ability to throw the outside stimulus due to repeated helplessness, they want to kill themselves.

## 7. Conclusions

Safety needs are the most fundamental needs for the human kind, which include personal security, financial security, and health and well-being. Safety is the major reason for mental disorders, such as anxiety, phobia, depression, and PTSD. The neural basis for safety is amygdala and LC/NE system, which can be regarded as a “safety circuitry,” whose major behavior function is “fight or flight” and “fear and anger” emotions, or conditioned learning for these emotions. Fear and anger are due to the safety needs, while joy and sadness are due to the physiological needs, which should come after safety needs in Maslow's hierarchy of needs. Fear and anger are two sides of one sword, for they will act in different directions: fear is to flight away from the danger and anger is to fight the danger away. They are all due to the stressful events: normally everything is as expected, and life is calm. When something unexpected happens, the individuals first feel scared and then blame the unexpectancy; this is the first safety check. Afterwards the individual will have a hedonic need to see if it fits their personal needs and get the happy or sad emotions. Finally, everything comes to an end, and people return to calmness or miss the lost things and worry for the uncertain bad things. So the emotional rainbow (or emotion flow)* fear-anger-happiness-sadness-missing* constitutes our emotions in everyday life.
